# Economic evaluation of healthcare-associated infection prevention and control in long-term care: a systematic review protocol

**DOI:** 10.1186/s13643-022-02128-7

**Published:** 2022-12-03

**Authors:** Eric Nguemeleu Tchouaket, Katya Kruglova, Idrissa Beogo, Drissa Sia, Stephanie Robins, Emilie Bélanger, Maripier Jubinville, Catherine Séguin, Kelley Kilpatrick, Sandra Boivin, Josiane Létourneau

**Affiliations:** 1 Department of Nursing, Université du Québec en Outaouais, St-Jérôme Campus 5, rue Saint-Joseph, Office J-2204, Québec, J7Z 0B7 Canada; 2 School of Nursing, Faculty of Health Sciences, University of Ottawa, Ottawa, Ontario Canada; 3 Ingram School of Nursing, McGill University, Montréal, Québec Canada

**Keywords:** Healthcare-associated infection, Infection prevention and control, Long-term care facilities, Economic evaluation, Discount, Cost, Effectiveness, Benefit, Systematic review

## Abstract

**Background:**

Given the high risk of contracting a healthcare-associated infection in long-term care facilities, infection prevention and control are essential for the quality of care and safety of residents and staff. To develop more effective infection prevention and control interventions in long-term care facilities, it is important to assess the cost-effectiveness and cost-benefit of existing interventions. There are only a few reviews on this subject, but these are not recent and most do not perform an economic evaluation. Moreover, none uses a discounting approach which limits inter-study comparison. To address these gaps, we will conduct a systematic review of economic evaluations related to healthcare-associated infection prevention and control in long-term care facilities using a discounting approach.

**Methods:**

We will query MEDLINE, Embase, Web of Science, Cochrane, CINAHL, EconLit, JSTOR, and Scopus, as well as the gray literature databases CORDIS and ProQuest. We will include quantitative studies that evaluate four clinical best practices associated with infection prevention and control (hand hygiene, hygiene and sanitation, screening, basic, and additional precautions) and use at least one of five economic analyses (cost-effectiveness, cost-benefit, cost-minimization, cost-utility, cost-consequences). Primary outcomes will include net cost savings, incremental cost-effectiveness ratio, incremental cost per quality-adjusted life year, and incremental cost per disability-adjusted life year. Two co-authors will independently screen and select articles, extract data, and assess the quality of selected articles using the Scottish Intercollegiate Guidelines Network criteria, the Economic Evaluation criteria, and the Cochrane criteria for economic evaluation. Extracted data will be synthesized, and values will be adjusted to 2022 Canadian dollars using the discount rates of 3%, 5%, and 8%.

**Discussion:**

Information obtained through this systematic review may help researchers and policy makers make more efficient use of limited healthcare resources to ensure the safety and quality of long-term care.

**Systematic review registration:**

Research registry ID: reviewregistry1210.

**Supplementary Information:**

The online version contains supplementary material available at 10.1186/s13643-022-02128-7.

## Background

Healthcare-associated infections (HCAIs) present a public health concern as they generate extra treatment costs, reduce quality of life, and increase the risk of morbidity and mortality [1–3]. HCAIs in long-term care facilities (LTCFs) may differ from those in acute care settings due to the complexity of illness management in the elderly, where infections may present atypically and may coexist with cognitive impairment [4]. The definitions of HCAIs specific to LTCFs have been published by Infection Prevention and Control Canada [5]. When applying these definitions, the following conditions must be met: (1) “signs and symptoms must be new or acutely worse than the resident’s baseline,” (2) “non-infectious causes should be considered first,” and (3) “identification of an infection should be based on both clinical presentation and diagnostic testing” (4). An infection can be attributed to an LTCF if it was not present on admission to an LTCF and if its onset occurred at least 48–72 h after admission (5).

LTCF residents are particularly vulnerable to HCAIs due to host risk factors such as multiple comorbidities and age-associated changes in immunity, as well as risks inherent in living in congregate environments [6]. Colonization of residents with antimicrobial-resistant pathogens and a transfer of residents between long-term and acute care settings confer additional challenges for controlling infections in LTCFs [6]. These factors contribute to high infection rates among residents, with those over the age of 65 being disproportionately affected [7]. An estimated 1.6–3.8 million infections occur in LTCFs in the USA each year resulting in 388,000 deaths [7]. The HCAI rate in Canadian LTCFs approximates 5–6/1000 resident days [8].

A 2020 systematic review listed the respiratory and gastrointestinal tracts as the two most common sites of infection among LTCF residents [9]. The most frequent etiological agents of outbreaks attributed to a single pathogen were influenza viruses and group A *streptococcus*. Other common culprits of outbreaks include noroviruses, *Salmonella* sp., *Clostridium difficile*, *Escherichia coli*, *Streptococcus pneumoniae*, and coronaviruses [10]. The newly emerged coronavirus, SARS-CoV-2, has exacted a heavy toll on LTCFs, as about 40% of all deaths due to COVID-19 worldwide occurred among LTCF residents between the beginning of the pandemic and January 2021 [11].

Given the high risk of contracting an HCAI in LTCFs, infection prevention and control (IPC) is essential for the safety of both residents and staff. Standard IPC measures encompass four clinical best care practices (CBPs) applicable to all settings: (1) hand hygiene, (2) hygiene and sanitation of surfaces and equipment, (3) screening on admission of residents who are either carriers or at-risk, and (4) basic and additional precautions such as isolation and the use of personal protective equipment [12]. These four measures have been clinically validated and integrated into the guidelines of the World Health Organization (WHO), Health Canada, the Canadian Patient Safety Institute, and the U.S. Centers for Disease Control and Prevention [13–15].

Despite the effectiveness of IPC programs using CBPs [16, 17], they typically receive only a small fraction of the healthcare budget. In 2019, the Canadian government spent an estimated $264.4 billion on healthcare [18]. Of this expenditure, only 8.6% ($602 million) was earmarked for public health activities and management costs, which generally cover IPC. As nearly half of an individual’s lifetime healthcare expenditure is expected to occur after the age of 65 [19, 20], it is critical to have an accurate and up-to-date estimate of the economic value of IPC in LTCFs. This value can be obtained through an economic evaluation, which estimates the costs and consequences of an IPC intervention using one of the following analyses: cost-minimization analysis (CMA), cost-effectiveness analysis (CEA), cost-utility analysis (CUA), cost-benefit analysis (CBA), or cost-consequences analysis (CCA) [21–24].

Only a few reviews have focused on the economic evaluation of IPC in LTCFs. Greig et al. [25] summarized the etiology, mode of transmission, morbidity and mortality rates, and preventive measures of 75 outbreaks of enteric illness that occurred in LTCFs worldwide between 1997 and 2007. Of the 37% of reports that provided outbreak prevention recommendations, none performed an economic evaluation. Cohen et al. [26] assessed cost estimates from nine studies of measures aimed at preventing infection among LTCF residents and staff. Most studies reported cost estimates of additional staff time or increased use of disposable items (e.g., gloves) and cleaning supplies. Only four studies conducted a cost analysis—one CUA, one CBA, and two CEA—and all were dated from 1992 to 2009. Uchida et al. [7] synthesized the rates and risk factors of HCAIs in LTCFs from 24 studies published between 2001 and 2010. In addition to the outdated evidence, only one study gauged facility-related costs associated with an IPC intervention but did not carry out an economic evaluation. More recently, Lee et al. [27] evaluated the effectiveness of IPC interventions to prevent HCAIs in LTCFs with the intervention components classified as per the WHO manual (e.g., education and training, staffing, and bed occupancy). However, no search restriction was applied to the type of intervention or economic analysis, and a qualitative synthesis was completed due to the heterogeneity of methodologies. Lee et al. [9] undertook a qualitative synthesis of 37 studies that reported outbreaks of pathogens in LTCFs. The focus was to clarify the causes of outbreaks and the measures used to control them, and thus, no economic analysis was specified in the search strategy. Furthermore, Moralejo et al. [28] conducted a Cochrane review to assess the effectiveness of interventions aimed at improving adherence of healthcare workers to IPC guidelines in any care setting. Only three studies focused on LTCFs, and these assessed healthcare-associated colonisation with methicillin-resistant *Staphylococcus aureus* (MRSA) as well as adherence to guidelines that excluded screening. Moreover, no specific economic evaluation was incorporated into the search strategy. Lastly, no review of IPC within LTCFs used a discounting approach to adjust costs and benefits for the period over which they occurred [29], thus limiting inter-study comparison.

To address these gaps, we will undertake a systematic review to update the evidence on the economic value of IPC using CBPs in LTCFs. Specifically, we will evaluate the cost-effectiveness of IPC interventions across the four CBPs (hand hygiene, hygiene and sanitation, screening on admission, and basic and additional precautions) and five economic analyses (CMA, CEA, CUA, CBA, and CCA). Broadly, we will attempt to answer the following question: Using a discounting approach, what is the cost-effectiveness of the four CBPs related to HCAI prevention and control in LTCFs reported in 2022 Canadian dollars (2022 CAD)? Our systematic review will provide an understanding of how to assess the rate of return on investment (i.e., ratio between net benefit and cost of investment) or cost savings of HCAI prevention and control within LTCFs.

## Methods

### Theoretical framework

**Fig. 1:**
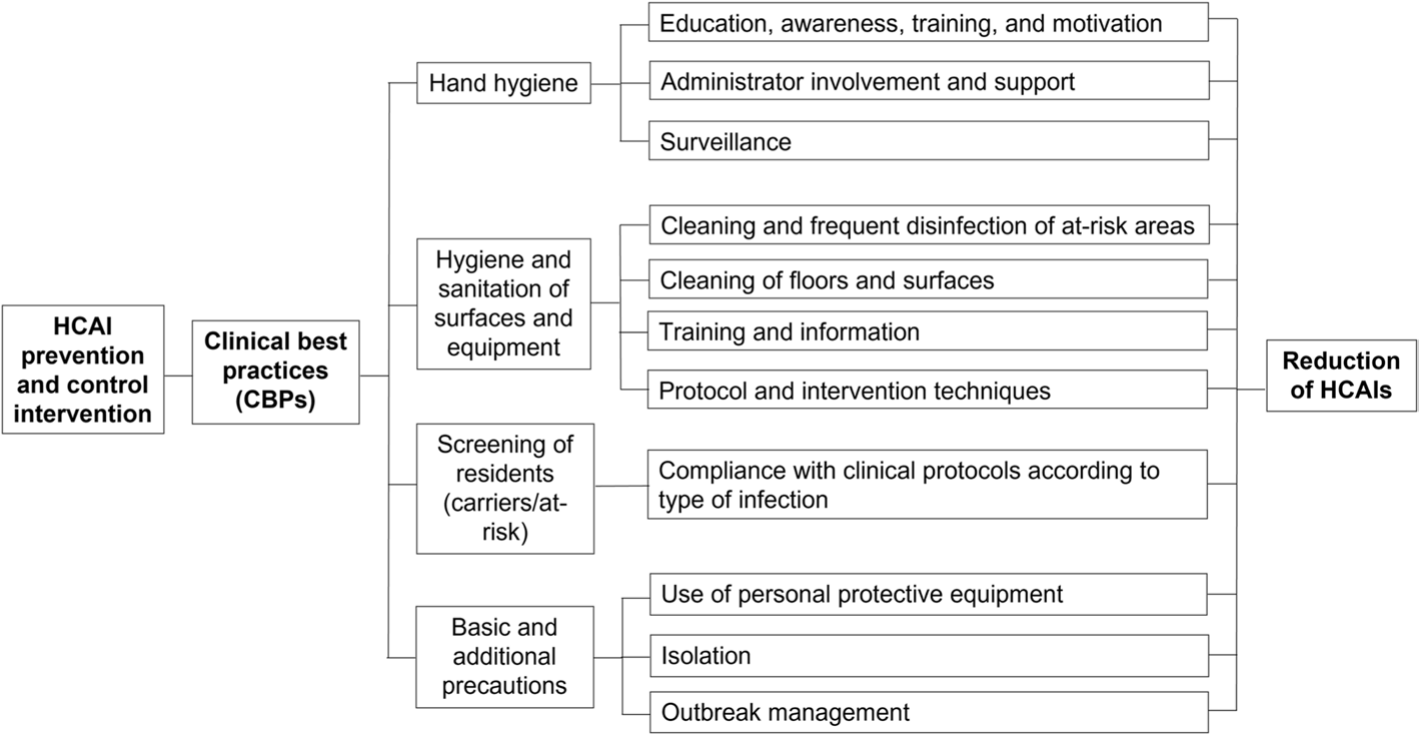
Theoretical framework based on clinical best practices (CBPs)

Figure 1 illustrates the theoretical basis of our systematic review, which was informed by the U.S. Institute for Healthcare Improvement’s framework of infection control interventions [12]. Within this framework, Resar and colleagues proposed a novel approach to improving the quality of health care, which is based on the use of care bundles, or small sets of evidence-based interventions implemented concomitantly to improve patient outcomes. We used this framework in our previous work [17, 30].

We will consider the following four CBPs related to HCAI prevention and control: (1) hand hygiene, (2) hygiene and sanitation of surfaces and equipment, (3) screening of residents according to established protocols, and (4) basic and additional precautions.

### Hand hygiene

Hand hygiene is the cleaning of the hands, wrists, and forearms either with water and soap or by applying a hydro-alcoholic or alcoholic antiseptic solution. Initiated by soaking the hands, this action continues until the hands are completely dry. The WHO estimates that hand hygiene may reduce HCAIs by 30–70% [31].

### Hygiene and sanitation of surfaces and equipment

The 2005 report “D’abord, ne pas nuire (First, do no harm)” by the Québec Ministry of Health and Social Services endorsed hygiene as a fundamental IPC measure [32]. Adherence to regular hygiene and sanitation of surfaces and equipment helps prevent the proliferation of pathogens. This action must be repeated with a frequency that is appropriate for infection prevalence rates at a specific site [33, 34].

### Screening of residents (carriers/at-risk)

Screening is the systematic testing of residents to identify potential carriers or those with a previously undetected infection. Residents without any signs or symptoms are considered potential carriers. Previously hospitalized residents are considered at-risk if they exhibit characteristic symptoms of an infection. Screening methods may differ depending on the type of pathogen and typically involve laboratory analyses to establish a clinical diagnosis. Urine, stool, and blood samples, along with nasal/oral and anal smears, may be obtained [35–38]. If a bacterial strain meets specific diagnostic criteria and is considered resistant, minimum inhibitory concentration tests are performed to determine the most appropriate treatment [35, 39].

### Basic and additional precautions

Additional precautions vary depending on the infection and may include the use of personal protective equipment, isolation, and contact precautions for residents with a known or suspected infection [40]. In the case of a major outbreak, CBPs must be intensively applied and can be supplemented by any relevant measures or resources over the course of the event [41, 42].

### Economic analysis

#### Analytical perspective, time horizon, and factors affecting costs

Appraisal of studies involving an economic analysis must consider three elements: the study’s analytical perspective, time horizon, and factors affecting costs, all while taking into account the resident’s baseline condition [43]. The analytical perspective focuses on the resident, an LTCF, or society at large, and determines costs that should be included in the calculations. For example, from the perspective of an LTCF, costs of family visit loss due to the resident’s isolation would not be included in the total resident costs. The time horizon refers to the period over which costs are to be estimated. Factors affecting costs encompass the disease stage and severity, comorbidities, risk factors, and the duration of stay at an LTCF [22, 43].

#### Types of economic analysis

We will consider five types of economic evaluations that are used to determine the effectiveness of IPC interventions: CMA, CEA, CUA, CBA, and CCA [21–24, 44]. CMA compares costs to determine the least expensive intervention while assuming identical outcomes. In a CEA, costs (in monetary units) and health benefits (in years of life gained) of an intervention are set against either those of another intervention or the status quo. The incremental cost is then divided by the number of years of life gained to obtain the differential cost-effectiveness ratio. In a CUA, health benefits are adjusted to signify the value of the years of life gained and expressed in quality-adjusted life years (QALYs). This approach estimates the differential cost-utility ratio, representing the additional cost required for an increase in QALYs. A CBA sets both costs and benefits to monetary units and derives the difference between the two in terms of a net gain or loss. Here, an intervention is contrasted with the status quo to measure the intervention’s return on investment. Lastly, in a CCA, no single cost-outcome ratio is calculated; instead, a table of estimated costs and all possible outcomes is compiled to allow decision-makers to judge the relative importance of the outcomes of interest.

### Research questions

An economic evaluation of HCAI prevention and control interventions takes into consideration the issues of prevention, the safety of care, and quality management. According to the Steven A. Finkler’s model, the cost of quality management integrates both the cost of investment in preventive measures and the cost of illness and care experienced [45, 46]. Finkler postulated that an investment in prevention can lead to an improvement in the quality of care once a certain threshold (or “optimum”) has been attained. Therefore, an economic analysis of the use of CBPs associated with HCAI prevention and control should address the following questions:

1. What are the costs of HCAIs in LTCFs?
2. What is the cost of investing in HCAI prevention and control based on CBPs in LTCFs?
3. What is the optimal break-even point to measure the return on investment or cost savings when comparing IPC intervention costs against potential benefits?

### Eligibility criteria

We will include studies that meet the eligibility criteria defined by the Population, Interventions, Comparators and designs, and Outcomes (PICO) framework, which is summarized in Table 1.

**Table 1:** Population, interventions, comparators and designs, and outcome framework

**Population**	
Geographic area	All countries
Establishment	Long-term care: nursing homes, assisted-living facilities, homes for the aged, retirement homes. *Excluded*: acute care.
Residents	All residents of LTCFs. *Excluded*: residents staying <72 h
Infections	Influenza viruses, noroviruses, *Salmonella* sp., Group A *Streptococcus*, *Sarcoptes scabiei*, *Clostridium difficile*, *Escherichia coli*, *Streptococcus pneumoniae*, Respiratory syncytial virus (RSV), *Legionella* spp., Parainfluenza viruses, *Mycobacterium tuberculosis*, Adenoviruses (epidemic keratoconjunctivitis), Hepatitis B virus, *Clostridium perfringens*, Rhinoviruses, *Chlamydia pneumoniae*, *Shigella* sp., Methicillin-resistant *Staphylococcus aureus* (MRSA), coronaviruses (SARS-CoV-2), rotaviruses, *Campylobacter* sp., trichophyton
**Interventions**	
Clinical best practices (CBPs)	Hand hygiene; hygiene and sanitation; screening on admission; basic and additional precautions. *Excluded*: antibiotics and any other medications.
**Comparators and designs**	Quantitative studies: controlled clinical trials, randomised controlled trials, cohort studies, longitudinal studies, follow-up studies, prospective studies, retrospective studies, cross-sectional studies, studies based on mathematical/statistical modelling, simulations. *Excluded*: qualitative studies, literature reviews (systematic reviews, meta-analyses, meta-syntheses, scoping reviews).
**Outcomes**	
Types of economic evaluation	Cost-minimization analysis (CMA), cost-effectiveness analysis (CEA), cost-utility analysis (CUA), cost-benefit analysis (CBA), or cost-consequences analysis (CCA). *Excluded*: technological assessments, purely clinical studies, and pharmacological studies.
Measures of economic evaluation	Costs estimates of CBPs, incremental cost-effectiveness ratio, incremental cost per quality-adjusted life year, incremental cost per disability-adjusted life year and the incremental cost-benefit ratio, net costs, and net cost savings

#### Type of population (P)

We will restrict study settings to LTCFs and only consider residents staying at these facilities for at least 72 h. Studies conducted in acute care settings (e.g., hospitals) will be excluded. We will include studies that examine the prevention and control of the infections that are denoted in the PICO framework (Table 1) [10]. All countries will be considered.

#### Type of intervention (I)

We will only include economic analyses of the four CBPs associated with HCAI prevention and control: (1) hand hygiene, (2) hygiene and sanitation, (3) screening on admission, and (4) basic and additional precautions. Studies that evaluate any practice(s) other than these four CBPs will be excluded.

#### Type of comparators and designs

The following study designs will be included: controlled clinical trials, randomized controlled trials (RCTs), cohort studies, longitudinal studies, follow-up studies, prospective studies, retrospective studies, cross-sectional studies, studies based on mathematical/statistical modelling, and simulations. Qualitative studies, systematic reviews, meta-analyses, meta-syntheses, and scoping reviews will be excluded.

#### Type of outcome (O)

Outcomes will include quantitative studies using CMA, CEA, CUA, CBA, or CCA, as well as studies using any combination of these analyses. Technological assessments, purely clinical studies, and pharmacological studies will be excluded. We will adopt the LTCF as the analytical frame and 1 year as the time horizon. The following measures of cost-effectiveness will be used: net cost savings (savings-costs), incremental cost-effectiveness ratio (ICER=effectiveness/costs), incremental cost per QALY, incremental cost per disability-adjusted life year (DALY), and incremental benefit-cost ratio (IBCR=savings/costs).

### Information sources

This systematic review protocol has been registered in Research Registry. The methods were developed in accordance with the Preferred Reporting Items for Systematic Review and Meta-Analysis Protocols (PRISMA-P) 2015 statement. A PRISMA-P checklist is included as Supplementary File 1.

Scientific articles will be retrieved via iterative exploratory searches in eight electronic databases: MEDLINE via Ovid, Embase, Web of Science, Cochrane, CINAHL, EconLit, JSTOR, and Scopus. We will also query the gray literature databases CORDIS and ProQuest. Database searches will be performed using the Boolean operators “AND” and “OR.” We will only include articles written in English or French and published between 1992 and 2022.

All co-authors, including two IPC program specialists (JL, SB), contributed to the determination of keywords. Search strategies were established in collaboration between all co-authors including an experienced librarian (CS) at the Saint-Jérôme campus of the Université du Québec en Outaouais. A CINAHL search strategy is presented in Table 2.

**Table 2:** CINAHL search strategy

No.	Queries
1	TI ('clostridium difficile' OR 'c difficile' OR 'c-difficile' OR 'c. difficile' OR c diff' OR 'c-diff' OR 'c. diff' OR clostrid* Carbape* OR "hospital acquired" OR "Cross infection" or nosocomial* OR iatrog*) OR AB ('clostridium difficile' OR 'c difficile' OR 'c-difficile' OR 'c. difficile' OR c diff' OR 'c-diff' OR 'c. diff' OR clostrid* OR Carbap* OR "hospital acquired" OR "Cross infection" OR nosocomial* OR iatrog*)
2	TI ('Urinary-Tract Infections' OR 'Urinary-Tract Infection' OR 'Blood-Borne Pathogens' OR 'acquired pneumonia' OR pneumonia OR 'associated pneumonia' OR flu OR cold) OR AB ('Urinary-Tract Infections' OR 'Urinary-Tract Infection' OR 'Blood-Borne Pathogens' OR 'acquired pneumonia' OR pneumonia OR 'acquired pneumonia' OR 'associated pneumonia' OR flu OR cold)
3	(MM "Urinary Tract Infections, Catheter-Related") OR (MM "Urinary Tract Infections+")
	(MM "Bloodborne Pathogens") OR (MM "Pneumonia, Pneumocystis") OR (MM "Pneumonia, Viral") OR (MM "Pneumonia, Aspiration") OR (MM "Community-Acquired Pneumonia") OR (MM "Pneumonia, Bacterial+") OR (MM "Healthcare-Associated Pneumonia") OR (MM "Pneumonia+")
4	TI (Gastrointestinalis OR Gastrointestinal OR gastroenteritis) OR AB (Gastrointestinalis OR Gastrointestinal OR gastroenteritis)
5	TI ('Haemophilus influenzae' OR 'Respiratory viruses' OR 'Influenza viruses' OR 'Parainfluenza viruses' OR Adenoviruses OR 'Escherichia coli' OR Shigella OR Rotaviruses OR Noroviruses OR Salmonella OR Rhinoviruses OR Chlamydia pneumoniae OR Enterovirus) OR AB ('Haemophilus influenzae' OR 'Respiratory viruses' OR 'Influenza viruses' OR 'Parainfluenza viruses' OR Adenoviruses OR 'Escherichia coli' OR Shigella OR Rotaviruses OR Noroviruses OR Salmonella OR Rhinoviruses OR Chlamydia pneumoniae OR Enterovirus)
6	(MM "Gastroenteritis+") OR (MM "Haemophilus Influenzae") OR (MM "Haemophilus Infections+")
	(MM "Respiratory Syncytial Viruses") OR (MM "Respiratory Syncytial Virus Infections") OR (MM "SARS Virus") OR (MM "Escherichia Coli") OR (MM "Escherichia Coli Infections") OR (MM "Shigella") OR (MM "Dysentery, Bacillary") OR (MM "Rotaviruses") OR (MM "Rotavirus Infections")
	(MM "Chlamydophila Pneumoniae") OR (MH "Salmonella Infections") OR (MM "Caliciviridae Infections")
	(MM "Legionella") OR (MM "Enterovirus Infections+")
7	TI ('COVID 19' OR 'corona virus' OR 'Respiratory syncytial virus' OR 'Respiratory infection' OR 'Respiratory infections') OR AB ('COVID 19' OR 'corona virus' OR 'Respiratory syncytial virus' OR 'Respiratory infection' OR 'Respiratory infections')
8	(MH "Carbapenem-Resistant Enterobacteriaceae")
9	(MH "Clostridium Infections+")
10	(MM "Iatrogenic Disease")
11	(MH "Cross Infection+")
12	(MH "Cross Infection+") OR (MM "Iatrogenic Disease") OR (MH "Clostridium Infections+") OR (MH "Carbapenem-Resistant Enterobacteriaceae")
13	#1 OR #2 OR #3 OR #4 OR #5 OR #6 OR #7 OR #8
14	(TI Staphylococcus aureus OR AB Staphylococcus aureus) AND (TI methicillin OR AB methicillin)
15	TI VRE OR AB VRE OR TI ERV OR AB ERV
16	(MH "Methicillin-Resistant Staphylococcus Aureus")
17	(TI Enteroc* OR AB Enteroc*) AND (TI vancomycin OR AB vancomycin)
18	(MH "Vancomycin Resistant Enterococci")
19	(TI Bacil* OR AB Bacil*) AND (TI Gram OR AB Gram) AND (TI Neg* OR AB Neg*)
20	(TI Bacil* OR AB Bacil*) AND (TI Gram OR AB Gram) AND (TI Neg* OR AB Neg* ) OR (MH "Vancomycin Resistant Enterococci") OR (TI Enteroc* OR AB Enteroc*) AND (TI vancomycin OR AB vancomycin ) OR (MH "Methicillin-Resistant Staphylococcus Aureus") OR TI ERV OR AB ERV OR (TI Staphylococcus aureus OR AB Staphylococcus aureus) AND (TI methicillin OR AB methicillin)
21	#10 OR #11 OR #12 OR #11 OR #12
22	#09 OR #16
23	TI ( Cost* OR econom* OR 'econom* analysis' OR efficienc* OR 'cost effect*' OR 'cost util*' OR 'cost benefit' OR 'cost consequenc*' OR 'cost effic*' ) OR AB ( Cost* OR 'econom* analysis' OR econom* OR efficienc* OR 'cost effect*' OR 'cost util*' OR 'cost benefit' OR 'cost consequenc*' OR 'cost effic*' )
24	(MH "Economics+")
25	#14 OR #15
26	TI ( controlled clinical trial* OR Randomized controlled trial* OR RCT OR blind OR case control* OR Case* OR cohort* OR longitudinal* ) OR AB ( controlled clinical trial* OR Randomized controlled trial* OR RCT OR blind OR case control* OR Case* OR cohort* OR longitudinal* )
27	(MH "Randomized Controlled Trials+") OR (MH "Clinical Trials+")
28	(MM "Case Studies") OR (MH "Case Control Studies+") OR (MH "Matched Case Control")
29	(MH "Prospective Studies+")
30	(MH "Prospective Studies+") OR (MM "Case Studies") OR (MH "Case Control Studies+") OR (MH "Matched Case Control") OR (MH "Randomized Controlled Trials+") OR (MH "Clinical Trials+")
31	#21 OR #22 OR #23 OR #24
32	TI (Hand* OR Aseptic* OR intervent* OR Program* OR Strateg* OR hygiene* OR Clean* OR control OR prevention OR screen* OR wash OR protect* OR isolation OR sanitation ) OR AB ( Hand* OR Aseptic* OR intervent* OR Program* OR Strateg* OR hygiene* OR Clean* OR control OR prevention OR screen* OR wash OR protect* OR isolation OR sanitation)
33	(MH "Handwashing+") OR (MM "Infection Control") OR (MM "Hygiene") OR (MH "Patient Isolation+")
34	(MH "Handwashing+") OR (MM "Infection Control")
35	(MM "Hygiene")
36	(MH "Patient Isolation+")
37	#17 OR #20 OR #25 OR #29
38	TI ('Long-Term Care' OR 'Assisted-Living Facilities' OR 'long-term-care facility' OR 'Homes for the Aged' OR 'Nursing Homes' OR 'nursing home' OR 'long-term care' OR retirement home) OR AB ('Long-Term Care' OR 'Assisted-Living Facilities' OR 'long-term-care facility' OR 'Homes for the Aged' OR 'Nursing Homes' OR 'nursing home' OR 'long-term care')

### Article selection

**Fig. 2:**
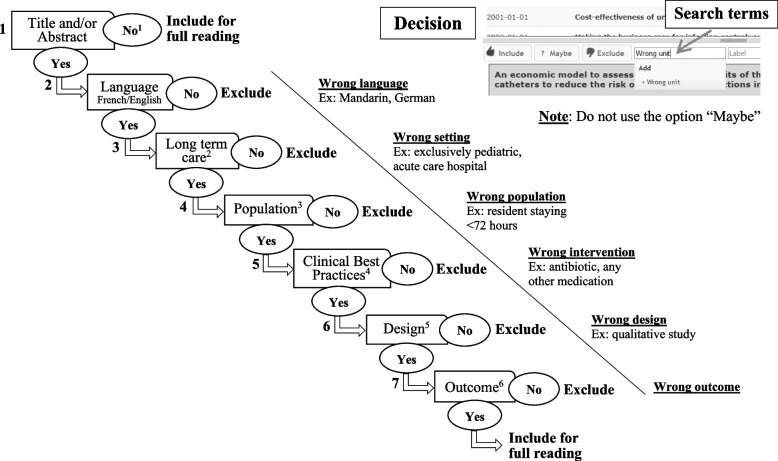
First round screening algorithm. Legend: ^1^Reference does or does not have a title and/or abstract. ^2^Long-term care: nursing homes, assisted-living facilities, long-term care facilities, homes for the aged, and retirement homes. Excluded: acute care (e.g., hospitals, clinics). ^3^Population: all residents of long-term care facilities. Excluded: residents staying <72 h. ^4^Clinical best practices: hand hygiene, hygiene and sanitation, screening, and basic and additional precautions. Excluded: antibiotics and any other medications. ^5^Design: quantitative studies (controlled clinical trials, RCTs, cohort studies, longitudinal studies, follow-up studies, prospective studies, retrospective studies, cross-sectional studies, studies based on mathematical/statistical modelling, simulations). Excluded: qualitative studies, literature reviews (systematic reviews, meta-analyses, meta-syntheses, scoping reviews). ^6^Outcome: cost estimates of CBPs, incremental cost-effectiveness ratio, incremental cost per quality-adjusted life year, incremental cost per disability-adjusted life year and the incremental cost-benefit ratio, net costs and net cost savings. Excluded: technological assessments, purely clinical studies, pharmacological studies

First, the research librarian will query the databases based on the established search strategies and create an Endnote database of retrieved articles. Duplicates will be identified and eliminated. All citations will be exported into the Rayyan web platform [47]. To improve reliability, prior to selection co-authors will screen the titles and abstracts of the same 10% of articles. Second, two co-authors (ETN, KKR) will independently screen the articles’ titles and abstracts by following an algorithm developed by our team (see Fig. 2). If any duplicates are identified, they will be excluded. If both co-authors consider an article eligible, it will be retained. If one of the reviewers deems an article ineligible, a third co-author will review the article’s title and abstract to arbitrate. If at least two of the three co-authors judge an article to be ineligible, it will be excluded. Third, after the first screening round, all equivocal articles will be reviewed by two other co-authors to reach a consensus. Fourth, the retained articles will be read in their entirety, and those fulfilling the eligibility criteria will be retained. Lastly, two IPC program specialists (JL, SB) will gauge the selected articles for their technical soundness and adherence to the PICO criteria.

### Data extraction

To extract data from selected articles, we will create an Excel spreadsheet based on the Consolidated Health Economic Evaluation Reporting Standards (CHEERS) [48]. We will extract the following information: author(s), year of publication, title and abstract, study objective, country, type of LTCF, study design, type of economic evaluation, sample size, population size, currency and adjustment year, time horizon, outcomes related to incremental cost, and funding sources. One co-author (KKR) will perform data extraction, followed by validation by the principal investigator (ENT). If evaluating an article’s content requires expertise in IPC, the article will be vetted by two IPC program specialists (JL, SB) to confirm the accuracy of the extraction.

### Assessment of quality of selected articles

We will assess the quality of selected articles by applying three tools that are commonly used to conduct economic evaluations. First, we will use the audit guidelines for economic evaluation studies recommended by the Scottish Intercollegiate Guidelines Network (SIGN) [49]. Second, we will apply the Economic Evaluation criteria developed by Drummond et al. [22]. We used these two tools in our previous work [23]. Third, to ensure compliance with the standards of the *Cochrane Handbook for Systematic Reviews of Interventions*, we will adhere to the Cochrane criteria for economic evaluation [50]. By employing these three tools, we will ensure the robustness of our assessment, since each tool considers different components of an economic evaluation that may differ within or between studies. We followed this approach in our previous systematic review [17]. The quality assessment will be conducted by two co-authors (ENT, KKR), and if a consensus is not reached, a third co-author will arbitrate. Articles will be classified into three groups based on their quality: (1) “high quality,” if the average score across the three tools is at least 80%; (2) “moderate quality,” if the average score is between 60 and 79.9%; and (3) “low quality,” if the average score is less than 60%.

### Data analysis and aggregation of results

For each intervention, we will tabulate the following parameters: costs, HCAI incidence reduction due to intervention, cost-effectiveness ratios, cost-utility ratios, and cost-benefit ratios. We will also report the year of calculation and the currency used.

Economic evaluations are context and time sensitive since resource use and costs are contingent on the setting, clinical practice, and exchange rate, which can generate high heterogeneity [51]. Therefore, to assess the level of heterogeneity across selected studies, we will calculate the *I*^2^ statistic with a value of 85% or greater representing substantial heterogeneity [52]. Furthermore, we will analyze extracted data using the dominance ranking matrix (DRM) developed by the Joanna Briggs Institute (JBI) [53]. We will follow this tool to classify interventions into three categories based on their favourability for decision making: strong dominance, weak dominance, and non-dominance.

All currencies will be converted into 2022 CAD [54]. Based on our previous work [49] and following the recommendation by Montmarquette and Scott [55], costs will be converted into 2022 CAD using the discount rates of 3%, 5%, and 8%. For each discount rate, we will calculate the median values of cost outcomes (in terms of HCAI reduction due to intervention) and incremental ratios (cost-effectiveness, cost-utility, and cost-benefit). We will conduct sensitivity analyses on the median values of cost outcomes, indicating the outcomes’ maximum and minimum values [56].

The Grading of Recommendations, Assessment, Development and Evaluations (GRADE) guidelines [57] will be applied to appraise the robustness of the proposed recommendations with respect to the efficiency of HCAI prevention and control using CBPs. If there is no information on inflation adjustment, we will contact the article’s corresponding author for clarification, and if not successful, we will assume the costs were adjusted to the last year of the data collection period.

### Ethics and dissemination

This study does not require ethical approval as it will not use individual patient data. This systematic review is included in the research program “Investir en prevention et contrôle des infections: Investir en PCI.” This program has been accepted by the Research Ethics Committee of the *Université du Québec en Outaouais* (Project #2022-1883). The results of this review will be published in a peer-reviewed journal and presented at scientific conferences.

## Discussion

### Implications

This protocol lays the groundwork for a systematic review aimed at synthesizing the current evidence on the cost-effectiveness of HCAI prevention and control interventions using CBPs in LTCFs. Findings of our systematic review can provide researchers and policy makers with evidence-based information, potentially allowing them to make a more efficient use of limited healthcare resources to ensure the safety and quality of long-term care.

### Limitations and strengths

One potential limitation of this systematic review is the exclusion of qualitative studies, purely clinical and pharmacological studies, and technological assessments. Another potential limitation is the consideration of only those interventions that use one or more of the four CBPs as defined by the theoretical framework. Furthermore, potential heterogeneity of extracted data may limit the generalizability of findings. Despite these limitations, our review will be the first to synthesize the existing knowledge on the cost-effectiveness of HCAI prevention and control in long-term care using a discounting approach. The application of three different tools to assess the quality of selected articles will enhance the robustness of our assessment. We will also maintain the rigor of our review by following the CHEERS checklist for data extraction and analyzing the data using the JBI's DRM and sensitivity analyses. Overall, the theoretical framework based on CBPs will guide us throughout all stages of the review, helping us maintain methodological congruence between the study’s objectives, data collection and analysis, and aggregation of results.

## Supplementary Information

**Additional file 1.** PRISMA-P 2015 Checklist.

## Data Availability

Data sharing is not applicable to this article as no datasets were generated or analyzed during the current study.

## Abbreviations

CBA: Cost-benefit analysis
CBP: Clinical best practice
CCA: Cost-consequences analysis
CEA: Cost-effectiveness analysis
CHEERS: Consolidated Health Economic Evaluation Reporting Standards
CMA: Cost-minimization analysis
CS: Catherine Séguin
CUA: Cost-utility analysis
DRM: Dominance ranking matrix
DS: Drissa Sia
EB: Emilie Bélanger
ENT: Eric Nguemeleu Tchouaket
HCAI: Healthcare-associated infection
IB: Idrissa Beogo
IBCR: Incremental benefit-cost ratio
ICER: Incremental cost-effectiveness ratio
IPC: Infection prevention and control
JL: Josiane Létourneau
JBI: Joanna Briggs Institute
KK: Kelley Kilpatrick
KKR: Katya Kruglova
LTCF: Long-term care facility
MJ: Maripier Jubinville
MRSA: Methicillin-resistant *Staphylococcus aureus*
PICO: Population, Interventions, Comparators and designs, and Outcomes
PRISMA-P: Preferred Reporting Items for Systematic Review and Meta-Analysis- Protocols
QALY: Quality-adjusted life year
RCT: Randomized controlled trials
RSV: Respiratory syncytial virus
SB: Sandra Boivin
SIGN: Scottish Intercollegiate Guidelines Network
SR: Stephanie Robins
WHO: World Health Organization
